# Inconsistencies in Quality of Life Data Collection in Clinical Trials: A Potential Source of Bias? Interviews with Research Nurses and Trialists

**DOI:** 10.1371/journal.pone.0076625

**Published:** 2013-10-04

**Authors:** Derek Kyte, Jonathan Ives, Heather Draper, Thomas Keeley, Melanie Calvert

**Affiliations:** 1 Primary Care and Clinical Sciences, University of Birmingham, Birmingham, United Kingdom; 2 Medicine, Ethics, Society and History, University of Birmingham, Birmingham, United Kingdom; 3 MRC Midland Hub for Trials Methodology Research, University of Birmingham, Birmingham, United Kingdom; The University of Hong Kong, Hong Kong

## Abstract

**Background:**

Patient-reported outcomes (PROs), such as health-related quality of life (HRQL) are increasingly used to evaluate treatment effectiveness in clinical trials, are valued by patients, and may inform important decisions in the clinical setting. It is of concern, therefore, that preliminary evidence, gained from group discussions at UK-wide Medical Research Council (MRC) quality of life training days, suggests there are inconsistent standards of HRQL data collection in trials and appropriate training and education is often lacking. Our objective was to investigate these reports, to determine if they represented isolated experiences, or were indicative of a potentially wider problem.

**Methods And Findings:**

We undertook a qualitative study, conducting 26 semi-structured interviews with research nurses, data managers, trial coordinators and research facilitators involved in the collection and entry of HRQL data in clinical trials, across one primary care NHS trust, two secondary care NHS trusts and two clinical trials units in the UK. We used conventional content analysis to analyze and interpret our data. Our study participants reported (1) inconsistent standards in HRQL measurement, both between, and within, trials, which appeared to risk the introduction of bias; (2), difficulties in dealing with HRQL data that raised concern for the well-being of the trial participant, which in some instances led to the delivery of non-protocol driven co-interventions, (3), a frequent lack of HRQL protocol content and appropriate training and education of trial staff, and (4) that HRQL data collection could be associated with emotional and/or ethical burden.

**Conclusions:**

Our findings suggest there are inconsistencies in the standards of HRQL data collection in some trials resulting from a general lack of HRQL-specific protocol content, training and education. These inconsistencies could lead to biased HRQL trial results. Future research should aim to develop HRQL guidelines and training programmes aimed at supporting researchers to carry out high quality data collection.

## Introduction

Patient-reported outcomes (PROs), such as health-related quality of life (HRQL) are increasingly used to evaluate treatment effectiveness in clinical trials [1]. Patients value information on their HRQL [2,3] and may use it to inform complex healthcare decisions [4], for example, choosing between treatments offering similar survival rates [5], or selecting an intervention with the most acceptable side-effect profile [6]. HRQL trial results may inform decision-making in the clinical setting [7], support pharmaceutical labeling claims [8] and influence healthcare policy [9]. Thus, it is important that trialists take steps to ensure that their HRQL data are valid, reliable and free from bias.

Reducing the risk of bias is a prime consideration of trial design and common methods of improving the quality of trials may include appropriate randomisation and allocation concealment, adequate blinding, minimizing loss to follow up and the use of intention to treat analysis [10]. Other potential sources of bias may still arise, however, such as inconsistencies in the way outcome data are collected across study sites. To mitigate the risk of this kind of bias, PRO trial data should be collected using standardised methods [8]. Ideally, the same data collection processes should be applied at all study sites and across all study groups; with the standardised methods that will be employed during the trial clearly outlined in the study protocol, and communicated to research staff through in-house training and supporting trial documentation, for example, standard operating procedures (SOPs) [10].

It is of concern, therefore, that preliminary evidence, gained from UK-wide group discussions at quality of life training days run by the MRC, Midland Hub for Trials Methodology, suggests there are inconsistent standards of HRQL data collection in trials, and related trial protocol content, training and education is often lacking. Furthermore, researchers have reported problems when encountering HRQL data which raises concern for the well-being of the participant in some way (hereafter referred to as ‘concerning’ data), which may occur on collection of the questionnaire from the patient, or at the point of data entry. When faced with ‘concerning’ data - typically represented by extreme HRQL scores, or contained within unprompted additional comments recorded on the questionnaire - some researchers reported administering ad-hoc, off-protocol, co-interventions to help improve HRQL: for example, facilitating referral of a patient suffering with depression to a counseling service. Co-interventions, i.e. “any intervention other than the experimental maneuver that alters the frequency of a trial’s outcome of interest” [11], may lead to bias if they are administered differently across trial arms. Unless co-interventions in a trial are formally reported and the associated costs captured, under- or over-estimates of clinical efficacy and cost-effectiveness may result.

Any threat to the integrity of PRO trial data should be comprehensively investigated. Therefore, in the absence of existing research, we conducted a qualitative study to explore the experiences and opinions of research nurses and trialists involved in the collection and inputting of PRO data in UK-based clinical trials, with a specific focus on HRQL. Our objective was to investigate reported inconsistencies in HRQL data collection in clinical trials, to determine if they represented isolated experiences, or were indicative of a potentially wider problem.

## Methods

Our study employed a qualitative research design; semi-structured interviews were used to gather data from researchers involved in the collection and entry of HRQL data in clinical trials. A favourable ethical review was received from the West Midlands Research Ethics Committee (ref no 12/wm/0068). All participants gave written informed consent prior to taking part in the study. Interview discussions were digitally recorded, professionally transcribed (verbatim) and anonymised prior to analysis. The anonymised transcripts were stored electronically in accordance with the University of Birmingham ‘Code of Practice for Research’ and will be preserved in an accessible form for ten years prior to being securely deleted. The interviews and primary analysis were conducted by DK, supported by regular meetings with HD, JI and MC, primarily concerned with promoting reflexivity.

### Participants and Setting

Researchers were recruited across the following sites: one primary care NHS trust, two secondary care NHS trusts and two clinical trials units. These sites were selected to facilitate recruitment of researchers with a variety of professional backgrounds. All sites were based in the UK. During recruitment, information about the study was cascaded to all research nurses, research facilitators, data managers and trial coordinators at these sites, through their respective research management structures. Individuals were asked to register their interest with DK, who determined eligibility, provided additional information about the study and answered any further questions. Potential participants were deemed eligible if they reported prior experience of involvement in HRQL data collection or data entry within a clinical trial, with direct experience of handling HRQL questionnaires. We sought maximum variation with regard to trial experience (e.g. specialist area, length of experience, primary/secondary care setting) during recruitment, in order to capture viewpoints from a range of researcher roles. Recruitment continued within each distinct staff group until there was data saturation, i.e. no new data was provided by the most-recent batch of interviews from that group.

### Interviews

In-depth semi-structured interviews were conducted by DK, following Fielding’s guidelines [12]. A topic guide was developed (Interview Topic Guide S1), comprising open-ended questions that maintained a focus on the research aims, but which was sufficiently flexible to allow participants to raise different issues that were important to them. TK piloted questions amongst eight researchers, including three research nurses and five clinical trialists, who held trial management or senior research nurse roles in oncology, osteoarthritis or injury rehabilitation research. The final version of the topic guide included questions on: (1) the experience of the participant regarding HRQL data collection or inputting; (2) the HRQL-specific trial guidelines and/or training that participants had encountered during their research careers; (3) whether ‘concerning’ data had been encountered, and if so, how it was dealt with; and, (4) the challenges associated with HRQL data-collection and areas in need of improvement. The topic guide evolved alongside data analysis, ensuring that subsequent interviews explored themes arising from ongoing analysis. Therefore, the research remained participant-led and allowed emerging hypotheses to be tested and challenged in subsequent interviews [13]. All interviewees were sent a summary of their interview for checking. One participant suggested minor alterations regarding the way in which an administrative element of their trial had been interpreted. Corrections were made and were subsequently approved by the participant. All other interview summaries were approved without amendment.

### Analysis

Ongoing and iterative content analysis drew upon principles of grounded theory [14] and utilised both constant comparison [13] and deviant case analysis [15]. Interview transcripts were examined in depth by DK (immersion), prior to first cycle coding. A mixture of in-vivo, process and initial coding methods were utilised during the first cycle [16]. Focused, axial and theoretical coding was employed during subsequent cycles [16]. The aim was to construct a hierarchical network of themes, which captured the essence of the data and facilitated the development of a core theory. All interviews were analysed using the Dedoose (© 2011 SCRC) qualitative data analysis software. HD, JI and MC formally reviewed a subset of 10% of the transcripts to enhance the credibility and trustworthiness of coding and interpretation. Further, regular, meetings were held to discuss the emerging data. Decisions were made about which developing hypotheses needed to be tested in subsequent interviews, and proposed changes to the topic guide were agreed or rejected. Finally, HD, JI, MC and DK met to agree on the final themes and core theory.

## Results

### Characteristics of Interviewees

26 researchers were interviewed. Interviewees included research nurses, trial coordinators, data managers and research facilitators, and were drawn from all five of the recruitment sites in the study. The characteristics of the interviewees are presented in Table 1. Interviews lasted on average 38 minutes.

**Table 1:** Characteristics of interviewees.

**Participants**	**Characteristics**
**Research Nurses**	
Sample	n=16
Experience as a Research Nurse, *Mean* (*range*)*, in years*	6 (0.25-16)
Total Nursing Experience, *Mean* (*range*)*, in years*	18 (2-30)
Specialist Areas, *n* (*%*)	General medicine (n=7, 44%); Neurology (n=2, 12%); Orthopaedics (n=3, 19%); Oncology (n=3, 19%); Ophthalmology (n=1, 6%)
**Trialists**	
Sample	n=10
Research Roles, *n* (*%*)	Trial coordinator (4, 40%); Data Coordinator (4, 40%); Research Facilitator (2, 20%)
Experience in Research Role, *Mean* (*range*)*, in years*	2.3 (0.75-4)
Specialist Areas, *n* (*%*)	Gen medicine (4, 40%); Elderly care (1, 10%); Obstetrics (2, 20%); Orthopaedics (1, 10%); Oncology (1, 10%); Rheumatology (1, 10%)
**Total Sample**	**n=26; 15 Primary care (58%); 11 secondary care (42%); mean experience in a research role = 4.62yrs(range 0.25-16)**

### Overview of Themes

Interviewee responses generated four main themes: (1) apparent inconsistency in HRQL data collection, (2) identified difficulties in dealing with ‘concerning’ HRQL data, (3) a perception that HRQL data collection could be associated with considerable researcher burden and, (4) identification of deficiencies in HRQL data collection management and areas in need of improvement. 

### Inconsistency in HRQL data collection

Interviewees suggested that HRQL data-collection is inconsistently managed between, and within, trials, both in terms of the logistics of HRQL measurement and in the handling of missing data.

#### Logistical issues

Interviewees reported considerable variation in the way in which different individuals administered HRQL questionnaires within/between trials. For example, the levels of privacy afforded to trial participants whilst they completed the questionnaire appeared to vary within/between trials, depending on location and staff concerned.

“If they’re an in-patient… they’ve got time to sit and do it within their space and privacy. Within primary care if you’re in a GP surgery and you’ve got loads of clinics going on… I’d like to give him a private room to go into and say “please sit in there, please take your time, please do this”, and, but it doesn’t happen. Very often they actually will fill them out in reception area.” [participant *3; research* *nurse*]

“I do know colleagues that will fill them out and ask the questions while… you know, quite loudly, in front of their relatives and in front of, you know, not in a soundproof room or in a corner.” [participant 15; research nurse]

When the HRQL assessment was planned to coincide with a participant’s clinic appointment, there was inconsistency reported with regard to the timing of questionnaire completion and interviewees were unsure about the optimal timing.

“There’s no guidance on when to do the quality of life quite often, and I don’t know where it is best to be done.” [participant 3; research nurse]

“It’s hit and miss whether it’s before or after [the clinic appointment].”[participant 6; research nurse]

They also held differing views about the level of assistance that should be given to their participants during questionnaire completion. Some highlighted the importance of having a researcher present in order to answer the participant’s questions, whilst others advocated self-completion without advice.

“I give the person the questionnaire and then um ask [the] patient to fill it in themselves yeah, without me helping them.” [participant 1; research nurse]

“I firmly believe that… the patient is giving up their time to partake in our research, and that I should be available… while they’re in there. Because quite a few patients actually panic a bit about, ‘I don’t understand this question,’ or, you know, ‘is that right?’…” [participant 4; research nurse]

#### Missing data

Asking about the interviewees’ approach to identifying, and dealing with, missing HRQL data elicited a range of differing responses. Some researchers reported routinely checking completed questionnaires for missing data, some described both checking for missing data and also examining the content of the answers for scoring errors, whilst others did not check the questionnaires at all.

“We do sit there with the patient and just go through it before they leave, just in case [there is] anything they’ve missed out.” [participant 11; research nurse]

“We as research nurses always check the answers to see what they’ve put.” [participant 17; research nurse]

“I was taught, in, in my early years of research… you give that form to a patient in an envelope and they complete it and put it in, and you don’t look at it.” [participant 15; research nurse]

This variation in approach was also evident when interviewees discussed methods of dealing with missing data. There were not only significant differences between trials, there were examples of differing approaches adopted for different trial outcomes, and even opposing arms, of the same trial, some of which risked the introduction of bias.

“There are specific questions within the [study] questionnaire that we do need to be answered… if they’re sort of left off, then we have to try and get in touch with the participants, ring them and ask them those questions over the phone... if they’ve completed the… necessary questions but then they’ve left the quality of life [questions] blank, we don’t have to chase them for that… I struggle a bit with that… I think you should make as much effort to get the responses to those as you should to get responses to the primary outcome really.” [participant 20; trial coordinator]

“If they go into our control group… they’re not gonna have any personal contact, then [the questionnaire] will go back out in the post with just a letter, sort of, saying, ‘Oh, you accidentally missed out this one,’ and, er, and hopefully they’ll return it back… if they’re put into an exercise, erm, intervention, then they do get seen by [a research facilitator]. I’ll ask her to take the questionnaire with her… she’ll help them to fill it in.” [participant 25; trial coordinator]

### Dealing with ‘concerning’ data

Research nurses (and to a lesser extent data managers) discussed discovering, and subsequently dealing with, ‘concerning’ HRQL data, i.e. that which raised concerns about a trial participant’s well-being. Just over half (n=15, 58%) of our interviewees reported that they had encountered ‘concerning’ HRQL data at some point during their research careers (participants 2, 5, 6, 8, 10, 16-21 and 23-26). Interviewees were asked whether any particular HRQL information gave rise to concerns. The most common responses were; a significant reduction in quality of life, for example, evidence of low self esteem or depression, or an indicated risk of self-harm or suicide.

“The difficult bit is when you have a comment section at the end… some patients choose that to [describe] something that’s happened to them… sometimes about erm depression and if you’ve read that, then you should act on it.” [participant 5; research nurse]

“The sorts of things that I’ve found concerning have been when people… have done sort of quite long rambling sort of letters, talking about… the struggles that they’re going through, and erm that nobody’s doing anything and nobody’s helping them and what are they supposed to do and does something really bad have to happen… for anything to change… you know ‘it’s all hopeless, what’s the point’ sort of thing.” [participant 20; trial coordinator]

#### Recognition and frequency

Interviewees reported that ‘concerning’ data presented in three ways; (1) most frequently via low domain or aggregate questionnaire scores; (2) via additional information provided by their participants (commonly un-requested comments provided on the back of the questionnaire, or in accompanying letters); and (3) during conversations with the trial participant that were prompted by the act of filling in the questionnaire.

“A couple of times where measurable erm data… that the doctor had done, showed yeah, maybe a bit of a slight wobble but everything is okay. Whereas they’re - according to their erm quality of life questionnaire, they will sort of say oh god, it’s all awful and it’s all really getting worse… that was noted and the patient was called back in early.” [participant 17; research nurse]

“People will send their questionnaires back and sometimes they’ll attach a letter, or... they’ll write little comments on the back… there’s been the odd participant erm that’s sent something back that I’ve sort of read and been a little bit worried about them.” [participant 20; trial coordinator]

“When we did the questionnaire, she got quite upset… so I spent most of the time talking to her about what help she could, erm, get, and we, I went online and looked up the number of the Alzheimer’s Society, and things like that for her.” [participant 22; research nurse]

Researchers discussed the frequency with which ‘concerning’ data was encountered, variously reporting it as ‘every other week’ (participant 21; trial coordinator), ‘two or three percent’ (participant 23; data manager) or as approximately ‘five percent’ (participant 24; research nurse) of the total volume of HRQL data viewed. Some used less precise phrases, for example, ‘a couple of times’ (participant 17; research nurse), or ‘sometimes’ (participant 20; trial coordinator): 11 interviewees (42%) reported that they had never come into contact with ‘concerning’ HRQL data.

#### Action

The majority of interviewees (n=23, 88%) thought ‘concerning’ data always warranted some form of response. No consistent way of responding emerged, however, possibly because interviewees also reported that there were generally no instructions available on what to do in such a situation. They therefore reported experience of a range of responses including the following; calling the trial participant back into clinic for a further consultation, altering their medication, offering comfort and, most commonly, referral to their GP and/or to a specialist health professional (generally following prior consultation with the participant).

“One consultant called me back in because the patient had left [the HRQL questionnaire] with him… and he’d looked at it and was concerned at what he was seeing, and saying, ‘That’s not the patient that presented to me. We need to call them back in and we need to,’ you know, ‘talk to them again’.” [participant 15; research nurse]

“The patient came back... [they] were advised over the phone to just change the medication slightly and increase the frequency of drops and then the patient came in earlier and was seen.” [participant 17; research nurse]

“The patient was contacted… The surgery was contacted. And it has been notified to the GP as well that, ‘We received this piece of information from your patient X.’ So, erm, the GP was aware at that point… that this patient is in, in such a state.” [participant 19; research nurse]

#### No action

One interviewee reported taking no action when encountering data indicating poor HRQL. Another described a strategy that had been implemented in their trial which was aimed at avoiding the issue altogether, namely, removal at the outset of questions that were thought likely to lead to the generation of ‘concerning’ data.

“When they say they haven’t got a really good quality of life, you think, ‘Oh, dear,’ you know... not really a lot I can do about that… I’m just recording what the patient has said.” [participant 23; data manager]

“Initially, we did have... one section of questionnaires which was more about, sort of, depression, erm, and it was decided to remove those because we didn’t necessarily… like, if someone put particular answers, we’d have to deal with that… so we decided to remove those.” [participant 25; trial coordinator]

#### Reporting of actions

Interviewees were asked if concomitant medicinal interventions, administered as a result of encountering ‘concerning’ data, would be reported through existing trial mechanisms. Opinion was divided. Some thought this data would be automatically captured.

“[the co-intervention] would be entered in the, in the database of our trial… So, if, if the other person who’s gonna see the patient the next time is looking at the data, they will be collecting that information, they will have that beforehand, and relate it to the, the records in the surgery.” [participant 19; research nurse]

Others felt that some trials might find it difficult to fully track such interventions, especially if the period between follow-up clinics was long, or the trial involved postal questionnaires.

“You don’t tend to get any information, erm, coming back from the GP… And I think most patients, you probably won’t see them again because some of those questionnaires may be just a one-off… they might be filling the rest of them in and sending through the post. So, if it’s not a follow-up thing where you’re gonna see the patient again. You might not know… So that’s become an issue.” [participant 13; research nurse]

Two interviewees talked explicitly about whether non-medicinal interventions, for example referral for clinical psychology or counseling, would be reported/captured: there was uncertainty over whether this would occur.

“I doubt it whether a… it depends on the system... there may be referral, like, the doctor, the GP may say, ‘Referred to the counselor,’… I don’t know whether I’ll be able to see it. I don’t know.” [participant 19; research nurse]

### Researcher burden

Some interviewees reported feeling emotionally and/or ethically burdened by the task of HRQL data-collection. This sense of burden was usually reported in connection with dealing with HRQL-related participant distress, encountering ‘concerning’ data, or (predominantly for research nurses) attempting to resolve the tension between the two roles of ‘researcher’ and ‘health-care practitioner’.

#### Participant distress

Our researchers reported frequently having to deal with patients who were particularly emotional following HRQL measurement. Some therefore felt uncomfortable providing HRQL measures that included questions they knew often distressed patients

“Some of the patients get quite upset, especially when you ask them about anxiety and depression… I have had occasions where patients just burst into tears… it does tend to be quite an emotional thing for them to, to discuss, really.” [participant 22; research nurse]

“It’s almost reinforcing all the problems and some of them have got, you know, ‘is this affecting your finances’, you know ‘are you anxious and worried’ and it’s like ‘well yes I am,’... if they’ve been unwell, sick… you just sometimes feel like it’s the last thing they need to be doing.” [participant 5; research nurse]

“I feel really awful because I’m the one who’s handed them this questionnaire... I don’t want them to feel, you know, leave feeling depressed or… feeling a bit worried, because that’s not what I’m here for.” [participant 11; research nurse]

Interviewees also reported that they could be emotionally affected themselves, following such encounters.

“It looked, to me, as a… completely fed up and frustrated patient, ready to give up life… and I started shaking when I read it.” [participant 19; research nurse]

“Some of the more severe ones, or the more sad ones… you do go home and, and think about them.” [participant 21; trial coordinator]

“I’m reading what’s happening to these ladies and gentleman, and it’s, it is heartbreaking. And I have cried sometimes.” [participant 23; data manager]

Some reported feeling the weight of responsibility when having to decide whether, and how, to handle ‘concerning’ data. They described the difficulty in deciding whether or not to intervene in these situations, and suggested that they did not always feel prepared or trained to handle such decisions.

“I remember at a steering group meeting, saying… ‘Well, okay, how would you advise I make that decision?’ and being told, ‘Well, you’re not a clinician, you’re not supposed to be making that decision,’ and me, sort of, saying, ‘Okay, but you’re asking me to!’… So it’s difficult.” [participant 21; trial coordinator]

#### Dual-role tension and duty of care

There were also reports of burden associated with a perceived tension, for some interviewees, between their dual professional roles as health-care practitioners and researchers.

“We're told constantly… recruit, recruit, recruit... and for commercial trials you have to hit your targets… [but] you build up that very close relationship with people and yeah, it’s… they are patients first.” [participant 17; research nurse]

“You do feel that, erm, that contradiction between being a researcher and having to be quite, erm, detached and it’s data. And, then, a lot of the patients I know as people, and I’ve visited them.” [participant 21; trial coordinator]

The majority reported resolving this tension in favour of the ‘patient’ (the trial participant), identifying a perceived duty of care which directed them to consistently place the needs of the patient over and above those of the study. Interviewees tended to justify this position either by appealing to their personal values, or by invoking the obligations associated with their profession: for example, ‘make the care of people your first concern’, from the Nursing and Midwifery Council Code of Conduct [17].

“I have been in that position and I have been told that the study comes first [because] that’s my role… at the end of the day… that’s not how it works… I am [a] registered nurse… I have to act upon that as well.” [participant 8; research nurse]

“You have got this ethical dilemma between the research and the, and the patient, but your patient always comes first, so there shouldn’t really be an ethical dilemma.”[participant 9; research nurse]

“You have duty of care to that patient… Let other people worry about the massive numbers and the quality of the data… Your duty of care is there and then to that patient.”[participant 24; research nurse]

There was, however, one individual who held the opposite view: that their primary responsibility rested with the trial.

“I actually think you’ve got a duty to produce good research data… I know the impact of quality of life data… and I’ve seen it go through NICE [National Institute for Health and Care Excellence] and I know how important it can be.” [participant 15; research nurse]

This interviewee felt that their job was to ensure clean data and that it was the responsibility of those outside of the research study - the participant’s GP and regular health care professionals - to monitor and deal with HRQL-related issues, such as anxiety or depression.

“They’re in the system, they’re not just seeing a [research] nurse… I think some people feel they’re the only person that can pick it up, but if we all work as a team when we’re all doing our job, those issues should be picked up as well elsewhere.” [participant 15; research nurse]

These statements were discussed with other researchers in subsequent interviews, however, no other individuals supported this point of view.

### Deficiencies in HRQL trial management

Interviewees reported perceived deficiencies in trial management relating to HRQL data collection and suggested there were four areas in need of improvement; (1) the provision of adequate data-collection guidelines; (2) the implementation of training; (3) the effective transfer of information between the trial team, the research staff and the trial participants; and, (4) the attitude of trial management groups (TMGs) to HRQL as an outcome.

#### Guidelines

Interviewees reported they had been given little guidance either in the trial protocol, or in SOPs, on the administration of HRQL measurement, and there were no guidelines on dealing with ‘concerning’ data.

“I don’t think there’s an overall clarity about… using quality of life measures... [if the patient writes] additional information [on their questionnaire], how does that get recorded, how does that get fed back to the team? What happens… if you are concerned about somebody? What’s the process? What level should we get involved?...speaking to patients on the phone… what sort of things should we be checking out to make sure that they are actually okay?… these sorts of issues aren’t really covered anywhere.” [participant 20; trial coordinator]

Interviewees wanted guidelines in these areas, which they felt might be especially useful for inexperienced researchers. There was no consensus, however, on the optimal format for such guidance. The majority supported the reproduction of HRQL guidelines within the trial protocol, but some felt that the protocol was not always written in a language that was readily accessible to them, and instead suggested a role-specific appendix, or a separate SOP or work instruction.

“All that the nurses have got really is the protocol, which is… it’s more for the PI [Principle Investigator], basically, because it’s so in-depth… and there’s the patient information sheet which is a whittled down version of the protocol. And there’s nothing really in between… for the nurses. There’s no [specific] guidance for us… So I think something in the middle would be nice.” [participant 11; research nurse]

#### Training

Interviewees expressed discontent at what they felt was a general lack of in-trial HRQL training and wanted improvements in this area.

“I did not have any quality of life training, had no idea of the importance of quality of life questionnaires, until I went on this [external course]... we should have been aware, because it’s such an important part… I really do wish that we’d had this training right at the beginning.” [participant 4; research nurse]

Some felt that HRQL training should be delivered as part of existing site inductions, and others that an external study day (or half-day) would better suit their needs. Again, there was a feeling that training would be particularly useful for junior researchers. Interviewees were asked what elements they would like to see included in HRQL training, answers included; the purpose and importance of HRQL measurement, the optimal methods of administration, dealing with difficult situations, counseling distressed participants and dealing with, and reporting, ‘concerning’ data without introducing bias (participants 1-9, 11-13, 15, 17-20, 22-25).

#### Education of data collectors and trial participants

Interviewees reported that their trial participants would sometimes decline to answer HRQL questions that they regarded as overly intrusive (e.g. surrounding sexual activity) or struggle to answer sections they regarded as of questionable relevance to their situation (e.g. questions surrounding depression given to participants at low risk of the condition), which might result in missing data.

“The feedback you get is... ‘I can’t understand why they’re asking me this’…”[participant 4; research nurse]

“They’re a bit, ‘Oh, well, what relevance has this got to me having my hip done?’…”[participant 11; research nurse]

“Usually there’s a lot of, around the sexual health... part of the, of the form, they do miss off, especially... They just refuse to answer.” [participant 23; data manger]

Research nurses suggested that their participants would be more inclined to answer such questions if they were able to educate them about the relevance of the individual questions, and the purpose and importance of HRQL data generally, to the outcome of the study. Many nurses felt unable to do this, because they were rarely given this information by the TMG in the first place.

“We could have done with the training on the relevance of quality of life much, much earlier on... I think that would have helped explain the role of quality of life to patients as well, erm, because the feedback you get is… ‘I can’t understand why they’re asking me this,’… so, I think it’s very important that you educate the interviewer so that they can explain. [participant 4; research nurse]

Two possible reasons were suggested for this lack information transfer. First, that TMGs may believe the majority of research nurses did not want to be overloaded with information during trial training and those nurses who wanted to find out more about the purpose of HRQL measurement could consult the protocol.

“The only people that were explained stuff were the GPs... we would tell them what the study’s about... ‘This is the study.’ ‘This is what we’re trying to find out…’ But when it came to the nurses, it wasn’t deemed necessary... There was a, a copy of the protocol... to find out more information... you don’t want to bog them down... you want to make it seem as easy as possible.” [participant 10; trial coordinator]

Second, that TMGs may assume that HRQL measurement was entirely straightforward and therefore did not warrant explanation.

“Quality of life questions. They’re deemed as, 'Well, it’s self-explanatory. You just put it through and you ask the questions.' Erm and that, I know, happens.”[participant 10; trial coordinator]

#### Attitude to HRQL measurement

A number of interviewees suggested that HRQL measurement was not taken seriously enough by TMGs, stating that they felt HRQL was often regarded as an ‘add-on’ rather than a valued outcome. Several stated that improvements in HRQL guidance or training would only be effective if TMGs adopted an earnest attitude toward optimal HRQL assessment.

“I think you’d need to have the other things in place, that quality of life is taken seriously and is not just thought of as an add-on… and have a good training plan… so that everybody then who was on a trial had that information at the start, they had that training and they had right, this is our role, this is what we do in a situation like this. And this is our duty, I think we’d have to have all of that sorted out before that system would work and be effective.” [participant 20; trial coordinator]

## Discussion

This study provides insight into the experiences of, and issues faced by, research nurses, trial coordinators, data managers and research facilitators involved in the collection of HRQL data in clinical trials in the UK.

### Principal findings

Our results suggest there may be inconsistencies in the quality of HRQL data collection across, and within, clinical trials, which has the potential to adversely affect the reliability and validity of trial results. This variation appears to arise from sub-optimal standards of HRQL-specific trial protocol content, training and education.

Standardisation of data-collection processes is a fundamental aspect of trial design, which is aimed at reducing bias and measurement variability and maximizing data quality [10]. Interviewees in our study reported that both between-site and within-site standardisation was lacking, particularly with regard to logistical aspects surrounding: the timing of HRQL assessment (before/during/after the clinic appointment); the levels of privacy and assistance given to trial participants completing their questionnaires; and approaches to the management of missing data. HRQL assessment should ideally be undertaken at the same pre-specified time points in a trial, normally prior to clinic appointments, which can have an undue influence on PROs such as HRQL [18,19]. Our findings suggest that decisions regarding the timing of HRQL questionnaire completion can be ad-hoc and, in the absence of trial-level instructions, have the potential to vary between trial sites, risking bias. Similarly, our results suggest that the levels of privacy and assistance afforded to trial participants involved in HRQL measurement are also inconsistent across study sites.

Missing data can be a particular problem in trials with a HRQL outcome, and unlike some clinical outcomes, retrospective data capture may not be possible [20]. Methods to minimise missing data should be considered at the design phase of the trial and implemented in a consistent way [21]. Implementation of such methods were not universally reported by our interviewees and there were clear examples of questionable practice. In one trial, it was reported that missing clinical outcomes were actively pursued whereas absent HRQL questionnaires or items were not sought at all (participant 20). This approach may be potentially damaging to the trial as HRQL data is often not missing at random and may, therefore, represent a potential source of bias, especially when levels are high and differ by treatment group [20,22]. In another trial, it was reported by one interviewee (participant 25) that varying methods for retrieving missing HRQL data were employed across opposing arms of the trial, with face-to-face methods being used for the intervention group and postal methods for the control, again, such an approach may risk the introduction of systematic bias.

Our data suggests that inconsistency in HRQL data-collection methods may stem from a general lack of HRQL or PRO-specific protocol content, supporting trial documentation (such as SOP’s), and training and education. In the absence of such guidance, some interviewees felt they were left to find their own way of administering PROs, which perhaps explains the variation in methods employed. It is not clear why PRO data collection methods were not routinely included in the protocols of trials where HRQL was a primary, or important secondary, outcome. As far back as 1997, Fayers and colleagues [23] released guidelines for protocol writers, which included specific examples of text aimed at improving HRQL data-collection. Other authors have since produced guidelines that also address aspects of this area, although most are limited by a lack of systematic development or stakeholder involvement [18,19,21,24-33]. More recently, the US Food and Drug Administration [8] and the Centre for Medical Technology Policy [34] have published guidance documents containing recommendations geared towards the management of missing data and aimed at optimising the implementation of PROs such as HRQL. Moreover, these guidelines specifically promote the training and education of both trial researchers and participants as a key element of clinical trial quality control.

There are, however, some limitations with the current PRO guidance documents. Data-collection is generally not the focus of any paper, meaning that recommendations in this area can be sparse when they appear. In addition, there is no clear consensus in the literature, therefore, differing guidelines are spread across a number of publications [35]. This may, in part, explain why our findings suggest that recommendations are not filtering down to the level of local trial site personnel. Our interviewees also suggested the reason for this lack of information cascade might be that trial management teams did not always feel specific guidance or training was warranted, either because HRQL data-collection was deemed to be self-explanatory, or that the protocol would already provide all necessary information and further guidelines might overload research nurses with additional (and presumably unnecessary) content. Our research nurse interviewees disagreed with these sentiments, appearing to welcome more information on all aspects of HRQL measurement, which they felt was not always straightforward and could present a considerable emotional and/or ethical burden. They also highlighted that the protocols they generally worked with rarely contained HRQL-specific information that was adequate to support their needs, and were not always pitched at a level that was readily accessible to them. Finally, it is of course possible that HRQL-specific information *was* available in at least some trial protocols, but that our interviewees could not accurately recall its content. Evaluation of the quality of HRQL protocol content, and the extent to which relevant guidelines are internalised by trial staff, warrants further research.

Our other main finding surrounds the previously unreported phenomenon of ‘concerning’ data, which was described as data that raised concerns about the participant’s wellbeing. ‘Concerning’ data, although arising infrequently, was reported to present significant challenges for those who dealt with it, with little or no guidance provided by their TMG on how to manage it. Some research nurses reported feeling a degree of tension between their dual roles as a researcher and practitioner when faced with such data. They acknowledged a professional obligation to make any concern for the trial participant their first priority, but as researchers, they also recognised a duty to maintain the integrity of the trial. This dual-role tension has been discussed elsewhere [36-39]. It can be problematic, as it has been suggested that the need to collect ‘clean’ data and minimise withdrawals has the potential to disproportionately influence decisions related to the wellbeing of the participant [36]. This view was not supported by our data, with all but one informant reporting that they consistently prioritised the wellbeing of the participant over the needs of the trial. This behavior demonstrates a widespread endorsement by our interviewees of the first principle of ‘good clinical practice’ as outlined in European Union directive 2005/28/EC: “The rights, safety and well being of the trial subjects shall prevail over the interests of science and society”. [40] As our findings suggest, however, this approach was not without its problems. In the presence of ‘concerning’ data (and in the absence of appropriate guidance), several interviewees reported the provision of non-standardised co-interventions, administered, in good faith, to assist the trial participant in distress. Some interventions, especially those involving onward referral or non-medicinal treatments for example, may not be captured by standard trial reporting systems, which could lead to co-intervention bias [11].

### Strengths and limitations of the study

The strength of this study is its use of qualitative methods to provide an insight into the previously unexplored issues surrounding PRO data-collection in UK-based clinical trials, with a specific focus on HRQL. Our use of semi-structured interviews allowed the exploration of several ‘core’ topics with each of our interviewees, but still allowed scope to investigate novel themes as they emerged. The validation of interview summaries by our study participants, and the triangulation of analysis by four different researchers are further strengths and lend credibility to the data.

A limitation was that the interviewer had prior knowledge of some of the issues that were likely to be discussed, which could have influenced data collection. We attempted to mitigate for this through the use of a topic guide that encouraged the use of non-leading questions and through other credibility enhancing processes, including regular team meetings aimed at facilitating reflexivity, peer review of verbatim interview transcripts and formal triangulation of coding. The recruitment methods meant the participants in this study were self-selected. The results we have presented could be particular to this (UK-based) sample and may not be readily transferred to dissimilar groups and research contexts. The interviewees were, however, recruited from a variety of settings in both primary and secondary care, and were selected to capture a range professional backgrounds and levels of experience.

## Conclusions

This is the first study to investigate the views of research nurses, trial coordinators, data managers and research facilitators involved in HRQL data collection. Our findings suggest that there are inconsistencies in the standards of HRQL data collection in some trials, which may affect the reliability and validity of trial data and could lead to biased results. These inconsistencies may stem from a general lack of HRQL-specific protocol content, training and education within trials. We also found that research nurses, and to a lesser extent data managers, are sometimes exposed to HRQL data that cause them to become concerned for the wellbeing of the trial participant. Again, there appears to be a lack of protocol content and training on how to recognise and respond to such data. This lack of guidance risks the provision of co-interventions, which may remain un-reported and have the potential to introduce bias. Further research, using both qualitative and quantitative methods, and undertaken internationally, is needed to determine the extent of each of the problems highlighted in this study.

Our findings underline the need for improved guidance on PRO data collection in trials and clearer, more detailed, descriptions of how to collect and manage these data in trial protocols and SOPs. Ideally, current guidance documents should be supplanted with internationally endorsed consensus guidelines, specifically tailored to the promotion of best practice in PRO data-collection. In the meantime, trialists should utilise existing PRO and HRQL-specific data-collection recommendations [8,18,19,21,23-34] to inform the trial design process, alongside more general high quality protocol guidelines such as the SPIRIT 2013 statement [10,41]. Trial management teams have a responsibility to provide local site personal with protocol content and supporting trial documentation, alongside trial training and education, which aids optimal collection of both standard and ‘concerning’ PRO data, whilst minimising the risk of bias.

## Supporting Information

Interview Topic Guide S1 (PDF)
