# Supplementary material for: Inconsistencies in Quality of Life Data Collection in Clinical Trials: A Potential Source of Bias? Interviews with Research Nurses and Trialists
Source: PLoS One. 2013 Oct 4;8(10):e76625. doi: 10.1371/journal.pone.0076625 (PMC3790726; doi:10.1371/journal.pone.0076625)
Supplement: Interview Topic Guide S1 — (PDF) [file pone.0076625.s001.pdf]

The **M**ethodological and **E**thical Issues Associated with Health-Related **Q**uality **o**f **L**ife Measurement in Clinical Trials (**ME-QOL**).

Researcher Interview Topic Guide

Guidance notes to the interviewer

Note: *If the participant becomes distressed or unwell, the interviewer will adopt the following approaches, dependent upon the participant's wishes:*

1) If the participant wishes, the interviewer will suspend or terminate the interview, and will stay with the participant until they are feeling better.

2) If the participant has another person to provide care, at the request of the participant, the interviewer will either suspend the interview and leave the room, or will terminate the interview completely.

3) If the interviewer feels it is warranted, and if the participant agrees, he will provide the contact details of appropriate support agencies.

4) If the interviewer feels that there is reason to be concerned for the physical/mental health of a participant, he will inform the participant of his intention to take the appropriate action, e.g. call the GP.

Points to discuss with the participant prior to signing the consent form

- Recap on key information in the PIS
    - I will be recording this interview, so I have something to help me remember accurately what we talk about today, the only people who will hear the recording are myself and my supervisor, is this ok?
    - If there is anything you find you do not wish to talk about please let me know. I will aim to follow your lead in terms of what we discuss, but if we do stray on to a topic that you are not keen to talk about, tell me straight away and we can discuss something else.
    - We can stop the interview whenever you like. If you would like to take a break, or feel upset or unwell, please let me know and we will suspend or stop the interview entirely.

Participant and researcher sign consent form if participant still wishes to take part.

<u>Introduction to Interview</u>

Thank you for agreeing to take part in this interview. The aim of this interview is to discuss your ideas and experiences surrounding the way in which research trials measure a patient's quality of life. There are no 'right' or 'wrong' answers, we are interested in *your* views based on your experience of administering quality of life questionnaires as part of your research role. I am now going to start the recording.

Begin Interview

Main body of Interview

1) How much experience do you have of viewing quality of life information?

Prompts

- Roughly how often do you view participant quality of life data during a trial?
- For what purpose(s) might you view quality of life data during a trial?

2) As a nurse/data manager under what circumstances can you imagine acting (or feeling tempted to act) on the quality of life data viewed during a trial?

Prompts

- What kinds of actions might you take in these circumstances?
- What factors do you think might influence the decision of a researcher tempted to act on the quality of life data they may have viewed during a trial?

3) What mechanisms were available within the trials you have been involved in, for reporting any intervention undertaken in response to viewing a participant's quality of life data?

Prompts

- EITHER: So how would you have known what to do under circumstances like those we have just been discussing? OR: How did you know about these mechanisms? What did you think about their general usefulness?

4) In your opinion, what are the challenges of administering quality of life questionnaires in trials?

Prompts

- What professional tensions might arise when administering quality of life questionnaire in trials?
- How do those designing research need to respond to these tensions?

5) Some participants complete the quality of life questionnaire, but then write extra (unrequested) information either on the bottom, or on the back of the form. Why do you think they do this?

Prompts

- What kind of information do you think they might add?
- Have you witnessed this in practice?
- What are your thoughts on what should be done with this extra information?

6) In your opinion, what are the key training needs for researchers involved in handling/administering quality of life data?

Prompts

- To what extent have any of the things we've discussed today suggested areas where you feel more training may be required?

- Where do you think people in your role/doing you job turn to for support when they face tensions for which they do not feel adequately trained or briefed?

Post Interview – Debrief

- I have no more questions, but I'd like to give you the opportunity to say anything else about quality of life questionnaires, your experience of completing them, or anything else we've discussed today?
- *Outline what will happen next:* (1) the recording will be typed up and annonymised, then analysed alongside all the other interviews, (2) we will send you a summary of this analysis (unless you would prefer that we didn't) and will invite your comments. You do not have to comment on these results if you do not wish to.
- Finally, if you decide that you do not want what you have said today to be included in my research, you will need to tell me this within 2 working days – so by [*insert an actual day, according to timing of interview*]. After this it will be too late to withdraw as I will not be able to untangle what you have told me from what other people have told me.
- Thank you for taking part in the interview today.
